# Full recovery of elective orthopedic surgery in the age of COVID-19: an 8-month retrospective cohort study

**DOI:** 10.1186/s13018-021-02286-9

**Published:** 2021-02-24

**Authors:** Teng-fei Lou, Zun Ren, Zheng-hua Sun, Wei Wang, Cun-yi Fan

**Affiliations:** Orthopaedic Department, Shanghai Jiao Tong University Affiliated Sixth People’s Hospital, 600 Yishan road, Shanghai, 200233 China

**Keywords:** Elective orthopedic surgery, COVID-19, New normalcy, Shanghai

## Abstract

**Background:**

The coronavirus disease 2019 (COVID-19) pandemic has led to dramatic disruptions to orthopedic services. The purpose of this study is to quantify the reinstatement of elective orthopedic surgeries of our institution in Shanghai, China, and share our first-hand experiences of how this region is managing the post-outbreak period.

**Methods:**

The number of patients receiving elective orthopedic surgeries was analyzed in the timeframe of 8 months since the start of the pandemic (from January 20 to September 16) and compared with the patients receiving the same treatment during the same period in 2019. And a detailed workflow for handling patients about to receive elective surgeries in the COVID-19 post-outbreak period was described.

**Results:**

The number of the selective surgeries in the first 3 months only accounted for 31.72% of the same period in 2019 (*p* = 0.0031), and the ratio reached 97.47% when it came to the last 5 months (*p* > 0.9999). The selective surgeries even surpassed the pre-epidemic level in months 7 and 8. And the difference of the surgeries was not significant in the whole eight observed months between 2019 and 2020 (*p* = 0.1526). No health care providers or hospitalized patients in orthopedic departments in Shanghai have been infected nosocomially.

**Conclusions:**

Elective orthopedic surgeries have been fully recovered from the COVID-19 pandemic in our institution, and the new normalcy established during the post-outbreak period helped this region co-exist with the impact of the virus well.

**Trial registration:**

Retrospectively registered, registration number: ChiCTR2000039711, date of registration: November 6, 2020

## Background

The coronavirus disease 2019 (COVID-19) pandemic has led to dramatic disruptions to orthopedic services [1–4]. In response to the worldwide crisis, most units restricted or ceased elective procedures, fearing nosocomial infection and preserving finite resources on meeting this unprecedented challenge. It has been reported by several studies that the COVID-19 pandemic caused substantial reductions in orthopedic service delivery regarding to outpatient and emergency room visits, hospitalizations, surgical volume, and economic incomes, with more significant decline in elective than emergency services [3–6].

As the first outbreak country of the pandemic, China promptly took urgent and strict measures to contain the outbreak. The willing obedience and stringent compliance of the whole nation also played a crucial role. At present, the outbreak in China has been successfully controlled, and daily life, work, and production have comprehensively resumed across the whole country including Wuhan [7]. Undoubtedly, this tremendous achievement is promising and inspiring for the entire world, especially for those still struggling in the middle of the crisis.

Shanghai, a densely populated, traffic developed international metropolis, was one of the earliest cities hit by COVID-19. The regional healthcare system, which is acknowledged as one of the most excellent performing in China including orthopedic services, was also profoundly affected by this pandemic during initial period. Following the downturn of the initial crisis and the clearance of local cases, orthopedic services in Shanghai has been extensively reinstated and maybe has reached or even surpassed the pre-epidemic level, and a new normalcy has been established to co-exist with the impact of the virus. However, until effective vaccine or treatment against the virus are readily available, there remains a certain degree of risk in terms of disease resurgence. The phase II outbreak of the SARS in 2003 proved that even a single neglected case may exert a disastrous effect on this hard-won achievement by rapidly crippling a health-care system [8]. Therefore, constant vigilance is required during the fragile process returning back toward normalcy to guarantee the provision of sustainable orthopedic services.

The purpose of this study is to quantify the profound impact on the orthopedic selective surgeries in our institution, the largest othopedic center in China, and share our first-hand experiences of how this region is managing the post-outbreak period. We hope that the information offered by our research will be useful and inspire other orthopedic departments around the world.

## Methods

This study was approved by the ethics committee of our institution, and the informed consent was waived by this same committee.

This study was carried out in Shanghai, which is an international metropolis with over 24 million residents. The regional healthcare system not only serves local residents but also radiates the whole nation. Our institution was the largest othopedic center in China, with over 460000 outpatient clinic attendances and 4000 total joints procedures in 2019.

Anonymous data of selective surgical procedures, including total hip and knee arthroplasty, implant removal, carpal tunnel release, arthroscopic surgeries for reconstructions of cruciate ligament, and procedures for disc herniation were retrieved from our institution. Exclusion criteria included diagnoses of revision joint replacement, septic arthritis, fracture, pathologic fracture, infection, osteomyelitis, malignant bone tumors, and neurologic compromise. A number of patients receiving the abovementioned surgeries are reported in an 8-month period from January 20 to September 16, 2020 (February 29 was excluded because of the intercalary year), and compared with the same period in 2019. A month (counted as 30 days) was used as the unit of analysis.

Shanghai reported its first confirmed case infected with COVID-19 on January 20, 2020. The emergence of the first case and the outbreak reported by Wuhan made patients with less severe conditions start to decline their attendance spontaneously for both surgical procedures and consultations. After then, Shanghai launched the first level emergency response on January 24, 2020. Forceful measures taken by government included “stay at home orders,” travel restrictions, school suspension, mandatory requirement for wearing masks in public, closure of inessential businesses, cancellation of social activities, and aggressive contract tracing and early quarantine measures for infected individuals. Public hospital directives involved reduction or cessation of elective orthopedic procedures and outpatient visits, converting inpatient wards and operating rooms specifically for COVID-19 cases, redeploying staff to fever clinics and isolation wards, and dispatching volunteers to support Wuhan. The series of measures preserved bed capacity and medical supplies, limited interpersonal interactions, and optimized the allocation of care providers.

Up to September 16, a total of 954 confirmed cases (342 local cases and 612 imported cases) had been reported in Shanghai, of which 909 have been cured and discharged, and seven have died (0.73% mortality). Actually, there were no new local cases since March 5, in which day the emergency response was adjusted from the first level to the second level. And in May 9, the emergency response was further adjusted to the third level. The imported cases pose no threat to local residents because of the strict quarantine policy. As the initial crisis subsides, normal elective services are gradually recovering to the pre-epidemic level and a new normalcy has been established.

**Fig. 1:**
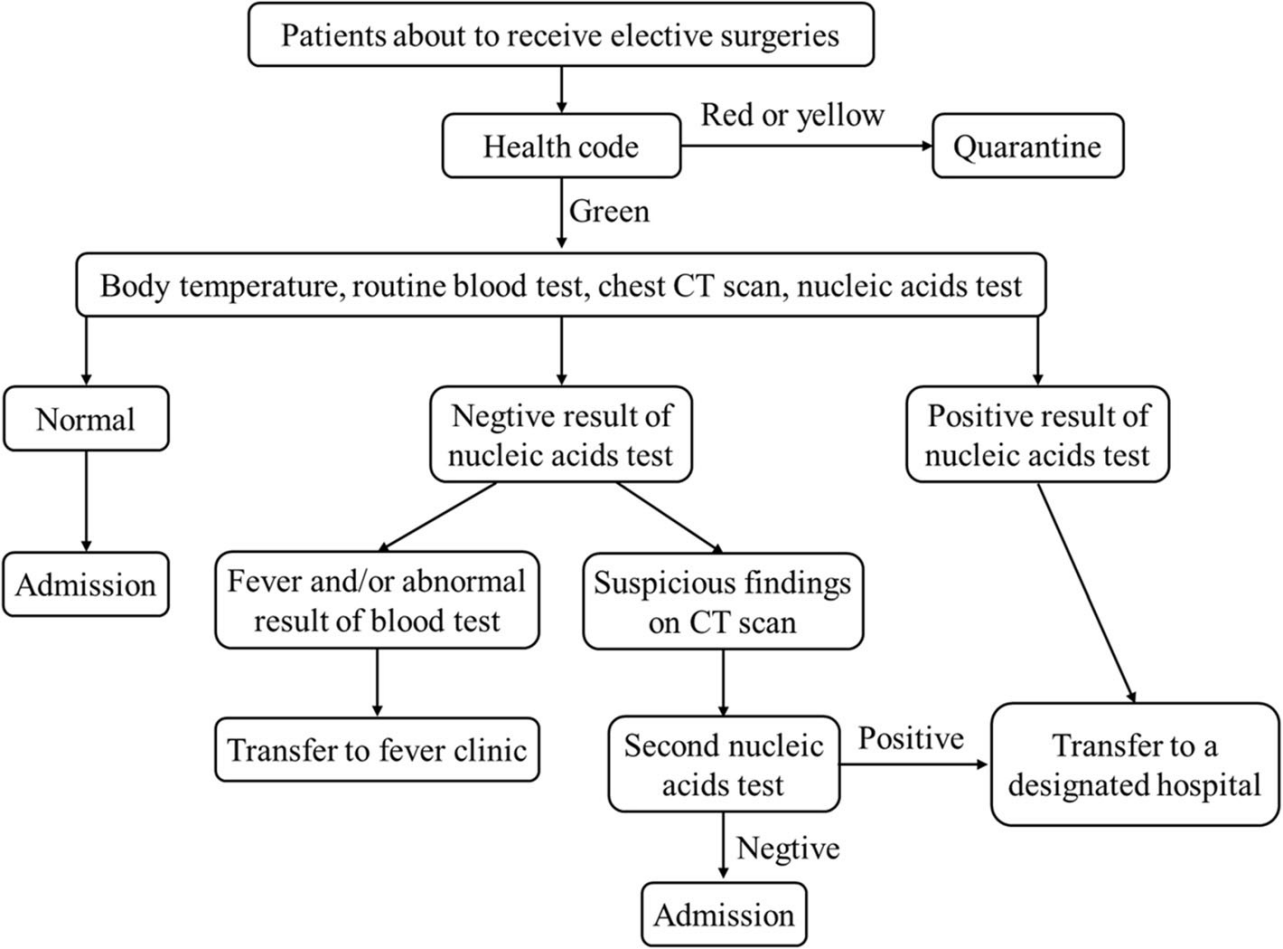
Flowchart for handling patients about to receive elective surgeries in the COVID-19 post-outbreak period

Comprehensive screening for fever, cough, sore throat, coryzal symptoms, anosmia, and travel and contact history is carried out at a triage station. It is worth mentioning that a health code system has been nation-widely used in China as a certificate for entry into public institutions including hospitals. This system indicates individual’s risk status based on basic health information and travel history. A green code has to be showed at triage station, which makes the screening procedure more efficient. Then, all the patients about to receive elective surgeries are required to undergo routine blood test, pulmonary CT scan, and COVID-19 RT-PCR test. Admission is permitted when all the results are normal. Negative results are valid for 7 days prior to admission. Patients with abnormal blood tests or fever but negative PT-PCR results are transferred to fever clinics for further diagnosis. Patients with suspicious CT results are asked to undergo the second RT-PCR test and antibody test. They will be admitted if the results remain negative; otherwise, they will be transferred to a designated hospital. Patients will be directly transferred to the designated hospital if the RT-PCR results are positive for the first test (Fig. 1).

Only one caregiver is permitted to enter the ward with a negative COVID-19 RT-PCR result and a normal body temperature. All the staff working in hospitals in Shanghai underwent COVID-19 RT-PCR test at least once between March and June, and no positive results were found. The staff were also required to undergo temperature and respiratory symptoms surveillance in the form of self-report through an online system. Stringent PPE precautions were carried out in early period. However, only mask is mandatory at present because of the relatively safe overall situation and the sound screening system.

Statistical analyses were performed using GraphPad Prism v6.0 (GraphPad Software, Inc., La Jolla, CA, USA). The level of significance was set below 0.05 (*p* < 0.05). Data recorded in the first 3 months and in the last 5 months of the 8-month period in 2019 and 2020 was compared by ordinary two-way ANOVA with Sidak’s multiple comparison test. The differences between the data over the observed 8-month period in 2019 and the corresponding period in 2020 were analyzed with the Mann-Whitney *U* test. The ratio between the number of different sub-specialties of 2020 and 2019 was calculated by months to investigate the speed and degree of resumption among different sub-specialties.

## Results

**Fig. 2:**
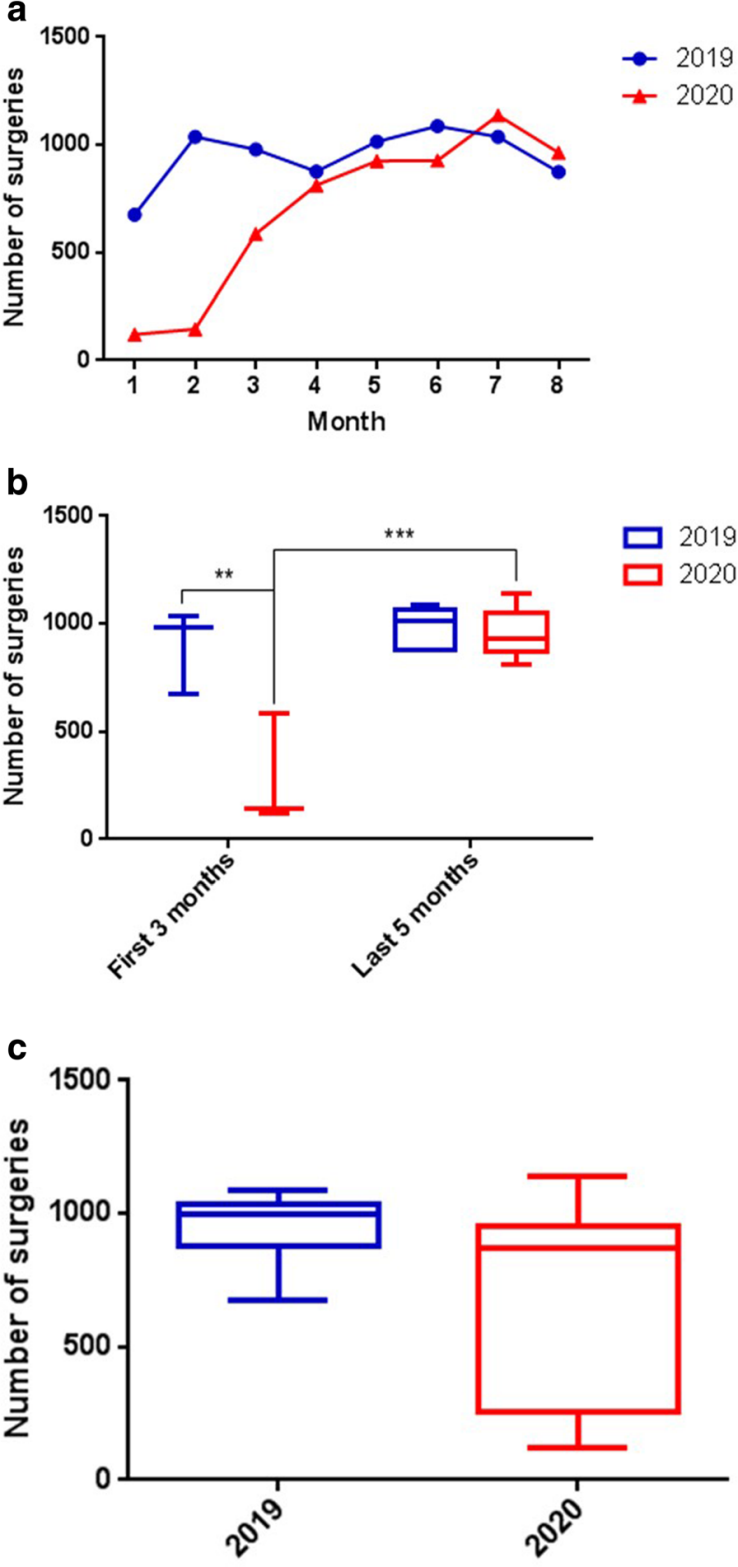
The number of the chosen selective surgeries from January 20 to September 16 in 2020 compared with the same period in 2019. **a** Trend of those surgeries during the 8 months. **b** The mean value of those surgeries 2020 vs 2019 (**p < 0.05, ***p < 0.01). **c** The mean value of those surgeries in the first 3 months and in the last 5 months in 2020 compared with 2019

**Fig. 3:**
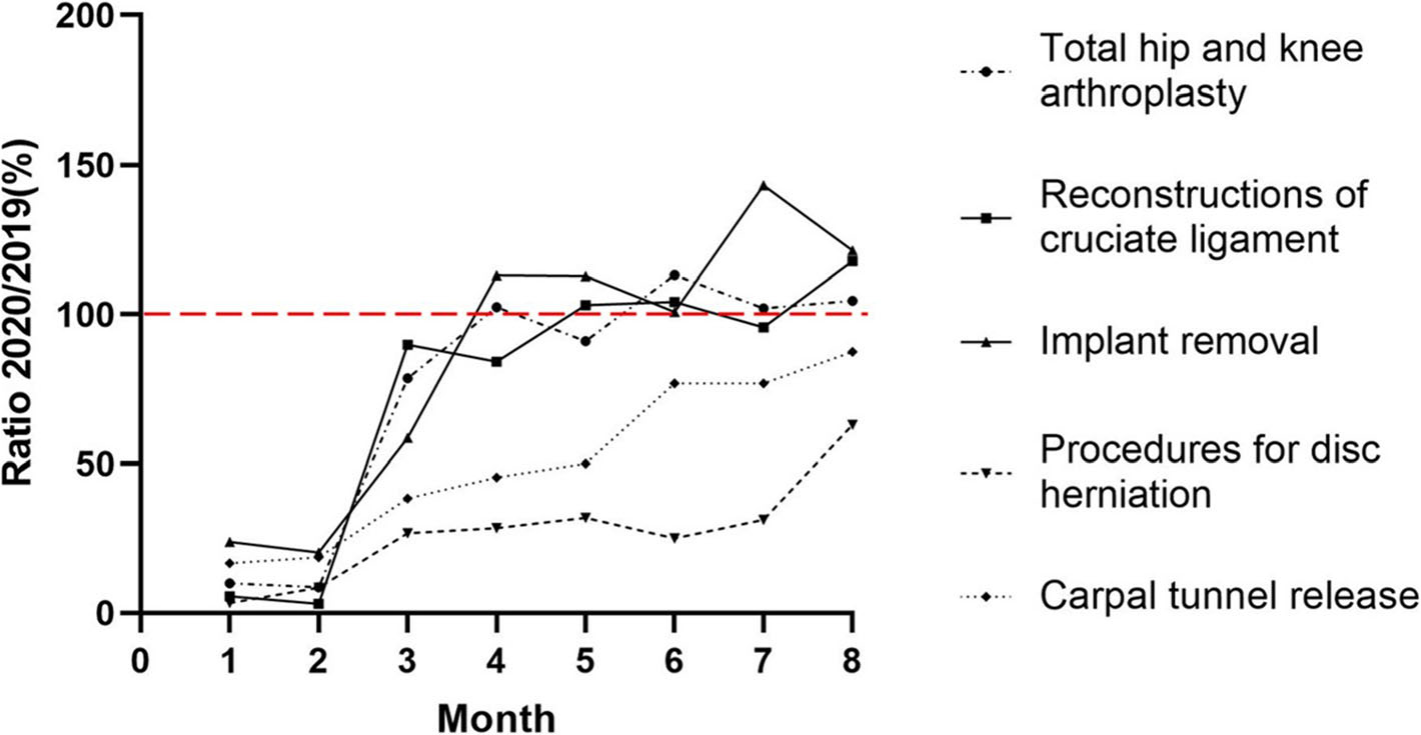
The trend of the ratio between the number of different sub-specialties of 2020 and 2019

The total number of those chosen selective surgeries decreased from 7584 in 2019 to 5622 in 2020 (25.87%, Table 1) in the whole 8-month period, but the tendency showed a rapid rise since month 2 (Fig. 2a). In month 4 of 2020, the number of the surgeries had reached 92.7% of the same period in 2019, and the number even surpassed the pre-epidemic level in months 7 and 8 (Table 1, Fig. 2a). The selective surgeries in the first 3 months in 2020 significantly dropped compared with the corresponding period in 2019 (*p* = 0.0031). The mean values of those surgeries in the first 3 months and the last 5 months were comparable (*p* = 0.9845) in 2019, while the latter was significantly increased compared with the former (*p* < 0.0006) in 2020 (Fig. 2b). Moreover, the selective surgeries in the last 5 months in 2020 reached the same level compared with the corresponding period in 2019 (*p* > 0.9999) (Fig. 2b). And the difference of the surgeries was not significant in the whole eight observed months between 2019 and 2020 (*p* = 0.1526), as presented in Fig. 2c, indicating that the elective orthopedic surgeries have been fully recovered from the COVID-19 pandemic. With regard to the recovery condition of different sub-specialties, we found that total joint replacement, ligamentous reconstructions, and implant removal procedures all began to return to pre-epidemic levels since the fourth month. However, carpal tunnel release and procedures for disc herniation showed a slow recovery and were consistently below pre-epidemic levels (Table 2, Fig. 3).

**Table 1:** The surgeries by months comparing 2019 and 2020

Year	Month 1	Month 2	Month 3	Month 4	Month 5	Month 6	Month 7	Month 8	Total
2019	675	1038	979	877	1015	1088	1038	874	7584
2020	121	146	587	813	925	928	1138	964	5622
Ratio 2020/2019 (%)	17.93	14.07	59.96	92.7	91.13	85.30	109.63	110.30	74.13
	31.72%			97.47%					

This table contains number of the specific elective surgeries during the corresponding 8-month period in 2019 and 2020 (from January 20 to September 16). The ratio between the number of these surgeries of 2020 and 2019 by months and by the first 3 months and the last 5 months are presented

**Table 2:** The ratio between the number of different sub-specialties of 2020 and 2019 by months

Surgeries	Month 1 (%)	Month 2 (%)	Month 3 (%)	Month 4 (%)	Month 5 (%)	Month 6 (%)	Month 7 (%)	Month 8 (%)
Total hip and knee arthroplasty	10.00	8.68	78.55	102.44	90.96	113.11	101.99	104.43
Reconstructions of cruciate ligament	5.56	3.13	89.80	84.13	103.03	104.11	95.65	117.65
Implant removal	23.80	20.24	58.57	112.90	112.73	100.76	143.09	121.30
Procedures for disc herniation	3.33	8.47	26.82	28.47	31.90	25.00	31.29	63.01
Carpal tunnel release	16.67	18.75	38.46	45.45	50.00	76.92	76.92	87.50

So far, no health care providers or hospitalized patients in orthopedic departments in Shanghai have been infected nosocomially.

## Discussion

The transition from cessation of elective orthopedic surgeries to the recovery of pre-epidemic activity is confronted with many challenges, none more critical than blocking the further spread of the virus and ensuring the safety of both patient and staff. Several professional organizations have provided recommendations and guidelines on how best to manage selective operation patients during post-epidemic period [7, 8]. However, as far as we know, this is the first quantitative study reporting the full recovery of elective orthopedic surgeries in the age of COVID-19. We only took 3 months to obtain this achievement, which was ascribed to the early clearance of local cases and the rapid formation of a new normalcy.

However, the precautions we took to manage elective surgeries might be considered unnecessarily costly, overly rigid and time-consuming for a region that has cleared its local infected cases for months. However, the following facts make these precautions significant and indispensable. Firstly, the reduction or even clearance of new daily COVID-19 cases does not represent a victory but rather a quiet period which is attributed to the joint efforts from all aspects of society. Remaining vigilant and preventing or limiting the potential “second wave” is most paramount during the process of reinstitution, especially when the influenza season is approaching. Secondly, reinstating orthopedic services may unlock the enormous pent-up demand for both admissions and outpatient visits, and the caregivers frequently required by orthopedic patients further complicate the situation. Hospitals’ complex environment will formulate a breeding ground for infection if even a single confirmed case is overlooked. Thirdly, the possible false-negative results for RT-PCR tests means a certain proportion of asymptomatic or pre-symptomatic COVID-19 patients will be tested negative [9], and these patients could be potential drivers of viral spreading [10]. This necessitates the chest CT scan to improve the diagnostic accuracy [11]. Though the risk can never be eliminated, it can be effectively reduced by a stepwise strategy with a sound screening system, a combination of various diagnostic methods and appropriate personal protection. Fourthly, recognized risk factors causing unfavorable prognosis from COVID-19 infection include old age, obesity, and impaired immunity [12]. These risk factors are frequently confronted in arthroplasty populations. It was also reported that patients who developed COVID-19 showed a high complication and mortality rate after elective surgery [13]. Furthermore, the development and universal use of an effective COVID vaccine may reverse the situation and bring the whole health care system back to normal. However, the effectiveness of the COVID vaccine is still controversial, a large part of people remains suspicious and refuses to be vaccinated, and the production capacity of the vaccine is also a problem to be solved. The universal vaccination may still have a long way to go. Therefore, we are obliged to take every precaution that we can to prevent against the transmission of COVID-19 in the peri-operative period. Moreover, orthopedic procedures are prone to generate aerosol, raising the potential risk of viral transmission in operating theater. A stringent and robust screening system should be set up to mitigate the risks for operating room staff members.

Effective communication and comprehensive informing are also critical to assuage patients’ concerns and gain their cooperation. Enhanced consent in terms of the screening process, PPE utilization, accompany limitation policy, additional cost requirements, and the new risks they may face should be given to all the patents and their family members.

In the face of a pandemic on such a scale, measures and endeavors from medical systems can only reduce the risk of nosocomial infection and help the already infected cases, which is important but limited. Prompt policies and strict enforcement of authorities, along with the active cooperation and compliance of the general public play a role of prime importance in preventing the spread of the virus. In this way, most regions in China nipped the virus transmission in the bud.

This study has several limitations. In the first place, the data was retrieved from a single institution. However, we believe the results reflect the recovery of selective orthopedic surgeries in the whole region because of the uniformity of the regional policy. Moreover, our study did not contain all the elective orthopedic surgeries but only the most common ones from different sub-specialities. But these surgeries were also representative because they were highly susceptible to the pandemic [5, 8]. Finally, its retrospective nature, which is inevitable because of the nature of the research contents.

## Conclusions

We recognize that our experience may differ from that of other systems and global generalization of our guidelines will be scarcely possible [13–17]. However, we hope that the full recovery of elective orthopedic surgeries in our institution will bring hope to other colleagues still facing a severe pandemic situation. Through reasonable measures, the pandemic will eventually be brought under control just like what has happened in many countries, we also hope that the new normalcy described in this study will be of use and inspire other orthopedic departments in the post-outbreak periods.

## Data Availability

The datasets used and/or analyzed during the current study are available from the corresponding author on reasonable request.
